# The relationship between teacher support and positive emotions in Chinese higher vocational students: multiple mediating effects of procrastination behavior and interpersonal assistance

**DOI:** 10.3389/fpsyg.2023.1201864

**Published:** 2023-06-27

**Authors:** Junhui Wang, Cheng Zhou, Qiming Song, Fuxiao Xu

**Affiliations:** ^1^School of Early Childhood Education, Hangzhou Polytechnic, Hangzhou, China; ^2^School of Public Administration, Nanjing Normal University, Nanjing, China; ^3^Institute of Higher Education, Nanjing University of Information Science and Technology, Nanjing, China; ^4^School of Educational Sciences, Nanjing Normal University, Nanjing, China

**Keywords:** teacher support, positive emotions, procrastination behavior, interpersonal assistance, mechanisms of influence

## Abstract

**Background:**

In China, more than 5 million students enter higher vocational colleges each year, and the positive emotions of their students merits much attention.

**Purpose:**

This study aimed to explore the effect of teacher support on positive emotions among higher vocational students by further investigating the mediating role of procrastination behavior and interpersonal assistance.

**Methods:**

A questionnaire survey was conducted with 676 higher vocational students from Zhejiang Province, China. We used SPSS 26.0 for data analysis, which included correlation analysis, regression analysis and bootstrap-mediated effects tests.

**Results:**

Teacher support positively predicted positive emotions (*β* = 0.302, *p* < 0.001) and interpersonal assistance (*β* = 0.170, *p* < 0.001), while procrastination behavior negatively predicted interpersonal assistance (*β* = −0.161, *p* < 0.001) and positive emotions (*β* = −0.088, *p* < 0.01). Interpersonal assistance positively predicted positive emotions (*β* = 0.279, *p* < 0.001). This study found that teacher support positively predicted positive emotions; either procrastination behavior or interpersonal assistance independently mediated the relationship between teacher support and positive emotions. These two variables (procrastination and interpersonal assistance) also acted as a chain mediator between teacher support and positive emotions. The direct effect of teacher support and the mediating role of interpersonal assistance had a greater effect.

**Conclusion:**

The study deeply explored the effects of teacher support on positive emotions among Chinese higher vocational students and found that teacher support plays an important role in positive emotion management. At the same time, we found the key roles played by procrastination behavior and interpersonal assistance between teacher support and positive emotion, which could provide data support and decision-making reference for enhancing higher education students’ well-being and positive emotions. This study can be regarded as a case study of social support theory and demonstrates the applicability of the theory in the field of positive emotions of higher vocational students.

## 1. Introduction

In recent years, the positive emotions of adolescents have attracted much attention from countries and societies worldwide and have become the focus of many scholars’ research. As an important factor influencing mental health, positive emotions are a complex psychological phenomenon with multiple components, including pleasurable experiences, positive cognitive evaluations, satisfaction of needs and corresponding expression patterns, as well as pleasant or satisfying situational responses (Cohn and Fredrickson, 2009). Fredrickson’s (2004) broaden-and-build theory of positive emotions is a good illustration of the broadening and building function of positive emotions. Specifically, the broadening function is the ability of positive emotions to expand an individual’s attention, cognition, action and other transient sequences of thinking and behavior, allowing the individual to solve problems more efficiently and flexibly. The building function mainly involves the long-term physical, cognitive, social and psychological resource construction and accumulation brought about by positive emotions (Gao and Tong, 2010). At present, there is a growing body of research that suggests the importance of positive emotions for personal mental health and well-being. Positive emotions have been found to predict levels of well-being, making individuals more resistant to stress and more vital (Diener et al., 2020). Generally, individuals with positive emotions are more likely to maintain physical health, increase efficiency and productivity, and avoid psychological problems (Nelson et al., 2016) also found that positive emotions can increase creativity in the workplace by helping employees think in new ways and solve problems and that employees’ positive emotions are significantly related to their performance on the job. Simultaneously, positive emotions can even improve individual decision-making and judgment, creativity and productivity, as well as family relationships, career development, and marital relationships (Hirschi, 2009). In addition, individuals with positive emotions are more likely to form social relationships, and scholars have found that higher levels of self-perceived positive emotions mean that it is easier to expand relationships, increase communication, and maintain connections (Sels et al., 2021). In summary, positive emotions have a significant impact on individuals’ lives, not only in terms of promoting family harmony and individual health but also in terms of increasing employee productivity. Similarly, positive emotions also have important positive effects on higher vocational college students, as Fredrickson and Joiner (2018) found that positive emotions can directly promote healthy behaviors in college students. However, most previous studies have examined positive emotion as an independent variable and have not considered it as a dependent variable for more in-depth mechanistic analysis.

Given the importance of positive emotions on mental health and well-being, we used positive emotions as the dependent variable while using teacher support, procrastination behavior, and interpersonal assistance as independent or mediating variables. There are three reasons for designing the variables in this way: (1) Teacher support is considered an important element in the college environment and has a direct or indirect impact on students’ emotions and behaviors. A student who feels the care and concern of teachers may be more positive and comforted about various things in life, thus increasing positive emotions. As Huang et al. (2023) showed, teacher support has significant positive relationships with academic achievement, mental health and social competence. (2) Students’ procrastination behaviors may have a negative impact on their emotions. Procrastination behavior may result in students’ inability to complete tasks or achieve goals within the allotted time, which in turn may generate negative emotions such as feelings of loss and frustration, and subsequently, these negative emotions may further trigger more severe procrastination behaviors. As Klassen et al. (2008) showed, academic self-efficacy is a significant predictor of academic procrastination. (3) Interpersonal assistance and social network building are closely related to mental health and emotional management. Interpersonal assistance can provide emotional support that enhances students’ self-esteem and self-confidence, thus promoting positive emotions. Chang et al. (2020) found that good levels of interpersonal assistance in adolescents promote positive expectations about the future, maintain a high level of self-concept, perceive satisfaction with life and experience more positive emotions. Similarly, Froiland (2015) also found that with parental support, adolescents were more likely to experience positive emotions, whereas adolescents who were subject to parental psychological control (nonsupportive behaviors) had significantly negative emotions. In conclusion, we used teacher support, procrastination behavior, and interpersonal assistance as independent or mediating variables, and positive emotions as the dependent variable in this paper.

## 2. Research hypothesis

### 2.1. Teacher support and positive emotions

In terms of the social network of mutual support (Taylor, 2011), teacher support is a typical social support behavior for students that not only provides a feeling or experience of being cared for and respected but also has a beneficial impact on students’ psychological and physical health. More specifically, teacher support is the positive feedback that students perceive from teachers’ behaviors, including their concern, help and understanding. During learning or life. Babad (1990) classified the types of support from teachers’ behaviors as emotional support and learning support. In following House’s (1981) taxonomy of types of social support, one can classify social support from teachers as emotional support, instrumental support, informational support and assessment support. Studies have shown that perceived social support is inversely related to depression, anxiety, irritability, sleep quality and loneliness in individuals experiencing social isolation and social alienation during COVID-19 and that higher levels of social support reduce the risk of depression and improve sleep quality during isolation (Grey et al., 2020). In addition, social support can enhance health care workers’ self-efficacy and resilience (Labrague and De los Santos, 2020; Xiao et al., 2020), and social support (including teacher support, parental support, and friend support) also positively predicts positive emotional adjustment in university students (Han and Feng, 2020). Based on this, this study proposed the following:

*H1*: Teacher support positively predicts positive emotions in higher vocational students.

### 2.2. Teacher support, procrastination behavior and positive emotions

Teacher support and positive emotions may be directly related, and there may also be alternative relationship pathways. Procrastination behavior may mediate the relationship between teacher support and positive emotions. Steel (2007) suggests that procrastination is generally the behavior of an individual in delaying or avoiding a scheduled action or task despite the expected negative consequences, and it is a failure of self-regulation on the part of the actor (Mohammadi Bytamar et al., 2020; Zhang et al., 2022). Procrastination is common in the population, with approximately 15–20% of the general population having problems with procrastination (Harriott and Ferrari, 1996). For the student population, procrastination is also very common, with an estimated prevalence of 50–95% (Steel, 2007). Existing studies suggest that teacher support can reduce students’ procrastination behaviors (Yang et al., 2023). In situations where students feel supported and cared for by their teachers, they are more likely to actively face academic tasks and avoid procrastination behaviors (Zheng et al., 2023). When students reduce their procrastination behaviors, they are more likely to be academically and socially active, which eventually improves their positive emotions (Tice and Baumeister, 1997). Based on this, we proposed the following:

*H2*: Procrastination behavior mediates the relationship between teacher support and positive emotions.

### 2.3. Teacher support, interpersonal assistance and positive emotions

Interpersonal assistance is a dimension of social support on the Psychological Resilience Scale for Adults (RSA) (Friborg et al., 2003). As a major component of social support, teacher support and interpersonal assistance have a positive correlation and internal consistency, i.e., teacher support can positively influence interpersonal assistance (Wang and Eccles, 2012). Positive emotions, on the other hand, are subjective emotional experiences that are acquired as individuals’ interests and needs are met, which is a positive psychological resource. Existing research suggests that psychological resilience positively predicts positive emotions (Wang and Wang, 2013). In essence, psychological resilience is a characteristic of an individual’s stable ability to recover from adverse circumstances to normality or a psychological trait that allows for better development despite adversity. For this reason, we can consider interpersonal assistance an important component of psychological resilience, and more specifically, individuals can obtain help for or vent their emotions through meaningful interpersonal assistance (Hu and Gan, 2008). In general, students who are high in social–emotional skills usually acquire some interpersonal skills and are good at listening and negotiating conflicts constructively, i.e., there is a positive correlation between interpersonal assistance and positive emotions. This positive correlation both helps them maintain interpersonal relationships and makes them inclined to seek and offer help when needed (Wang et al., 2019). Teacher support can enhance students’ self-efficacy and learning motivation, which in turn increases their classroom engagement and positive emotions (Liu et al., 2018). Additionally, teacher support can lead to students’ healthy behavioral and emotional development, which helps them better deal with complex emotional states and social emotions (Jennings and Greenberg, 2009). In addition, teacher support can also facilitate the development of students’ interpersonal relationships and social networks, which can enhance their interpersonal support behaviors, and this is an important source of positive emotions (Henderson and Mapp, 2002). In other words, teacher support not only enhances students’ positive affect but also optimizes their interpersonal relationships and interpersonal assistance behaviors, which in turn promotes their positive emotions. Thus, interpersonal assistance plays an important mediating role in the relationship between teacher support and students’ positive emotions. In conclusion, interpersonal assistance plays an important mediating role in the relationship between teacher support and students’ positive emotions. Based on this, this study proposed the following:

*H3*: Interpersonal assistance mediates the relationship between teacher support and positive emotions.

### 2.4. Teacher support, procrastination behavior, interpersonal assistance and positive emotions

As mentioned earlier, teacher support not only reduces students’ procrastination behaviors but also enhances positive emotions. Likewise, high interpersonal assistance can also increase an individual’s positive emotions. Procrastination behavior and interpersonal assistance are closely related, and more specifically, procrastination behavior can affect an individual’s task completion time and schedule, thereby increasing the burden of interpersonal assistance and feelings of maladjustment. Existing research suggests that individuals who delay completing tasks often fail to anticipate the demands and expectations of the next task, which in turn decreases collective performance and drives other members away from these procrastinators, thereby reducing collective interpersonal assistance and support (Van Hooft and Van Mierlo, 2018). Other studies have also demonstrated that procrastination affects cognitive and affective states, negatively impacting not only the individuals themselves but also interpersonal relationships, causing pleasurable communication to be difficult and emotional exchanges to become unstable (Steel, 2007). In summary, the effects of teacher support on positive emotions are exerted primarily through procrastination behaviors and interpersonal assistance. That is, teacher support reduces procrastination behavior, which in turn increases interpersonal assistance, thereby increasing levels of positive emotions. It is inferred that procrastination behavior can negatively influence interpersonal assistance. As a result, the following hypothesis is proposed in this study:

*H4*: Procrastination behavior and interpersonal assistance act as a chain mediator in the relationship between teacher support and positive emotions.

Overall, despite numerous studies exploring positive emotions, most have focused on undergraduate students (Balkis and Duru, 2016; Hen and Goroshit, 2020), and few have explored higher vocational students. Simultaneously, existing scholars have mainly examined positive emotions as an independent variable and rarely as a dependent variable, nor have they analyzed the relationship between teacher support and the influence of positive emotions. Therefore, this study attempts to examine the mechanism of teacher support on positive emotions by investigating the separate mediating roles of procrastination behavior and interpersonal assistance and their chain mediating effect with a study of higher vocational students in China. This study focused on the mechanisms of influence between teacher support and positive emotions of higher vocational students, while also paying attention to the mediating effects of procrastination behavior and interpersonal assistance. The research can provide an important reference for decision making in emotional management and interventions by administrators of higher vocational colleges. Social support theory suggests that individuals who receive support and assistance from other people or groups can improve their well-being (Cohen and Wills, 1985; Uchino, 2009). In this study, teacher support and interpersonal assistance are specific forms of social support, while students’ positive emotions can be considered an indicator of mental health. Thus, the findings of this study are corroborated within the framework of social support theory. Conversely, the research could also confirm the applicability of social support theory in higher vocational students’ positive emotions and suggest the mechanisms by which teacher support and interpersonal assistance influence students’ positive emotions. In other words, this research can provide new data support and empirical cases for social support theory.

## 3. Research instruments, data, and methods

### 3.1. Research instruments

The study drew on existing well-established scales for data collection, with the variable “Teacher support” drawing on the Perceived School Climate Scale, PSCS (Jia et al., 2009). The scale had a KMO value of 0.770 in this study, and the chi-square statistic of Bartlett’s sphericity test reached a significance level of 0.1%, thus having good construct validity. For the variable “Procrastination behavior,” we use the Short General Procrastination Scale, SGPS (Stanton et al., 2002; Sirois et al., 2019; Zhang Y. et al., 2020), with a KMO value of 0.866 in this study, and the chi-square statistic of Bartlett’s sphericity test reached a significance level of 0.1%, thus indicating good construct validity. For the variable of “Interpersonal assistance,” we used the Psychological Resilience Scale, PRS (Connor and Davidson, 2003; Hu and Gan, 2008). The scale had a KMO value of 0.848 in this study, and the chi-square statistic of Bartlett’s sphericity test reached a significance level of 0.1%, thus having good construct validity. Regarding the “Positive emotions” variable, the Positive Affect and Negative Affect Scale, PANAS (Watson et al., 1988), was used in this study, with a KMO value of 0.739 in this study, and the chi-square statistic of Bartlett’s sphericity test reached a significance level of 0.1%, thus indicating good construct validity. All the scales are rated on a 5-point Richter scale, and the main items and the retest reliability coefficients are shown in Table 1.

**Table 1:** Scale items and retest reliability.

Variables	Scales	Items
Teacher support	PSCS α = 0.873	I can talk to my teachers about my problems
		My teachers care about me
		Teachers help students with problems
		Teachers help students with school problems
		Teachers believe I can do well
		Teachers work hard to get me to do well on tests
		My teachers make me feel good about myself
Procrastination behavior	SGPS α = 0.805	In preparing for some deadlines, I often waste time by doing other things
		I am continually saying I’ll do it tomorrow
		I often have a task finished sooner than necessary
		I generally delay before starting work I have to do
		I usually accomplish all the things I plan to do in a day
		I usually take care of all the tasks I have to do before I settle down and relax for the evening
		Even with jobs that require little else except sitting down and doing them, I find they seldom get done for days
		I often find myself performing tasks that I had intended to do days before
		I usually buy even an essential item at the last minute
Interpersonal assistance	PRS α = 0.809	I cannot find the right person to talk to when I’m not happy
		I have a friend of my own age who I can talk to about my problems
		I cannot find someone to talk to when I need help with a problem
		I have a habit of keeping things inside rather than talking to someone
		I will talk to someone when I am in trouble
		I do not want to talk to anyone when I’m upset
Positive emotions	PANAS α = 0.843	Interested
		Excited
		Strong
		Enthusiastic
		Proud
		Alert
		Inspired
		Determined
		Attentive
		Active

### 3.2. Data source

The survey was conducted using a convenience sampling method, and information was collected online through the Questionnaire Star platform (powered by www.wjx.cn). The questionnaires were distributed from February 21–March 16, 2023. To reduce common method bias, this study used procedural controls and statistical controls during data collection. The procedural control included the use of anonymous completion, emphasizing the confidentiality of the data to the subjects, and setting positive and negative questions; the statistical control method used the Harman one-way test for common method bias. The questionnaires were sent to the students through the online questionnaire link by the faculty of the higher vocational college, and the students voluntarily completed the questionnaires after receiving the link. The questionnaires were scored on a five-point Likert scale, and all data were entered into SPSS 26.0 for preliminary statistics. Finally, a total of 788 questionnaires were distributed, and 778 questionnaires were collected. We eliminated 112 invalid questionnaires based on response time and answer repetition rate and obtained a total of 676 valid questionnaires, for an effective rate of 85.8%.

The study subjects were divided by demographic variables as follows (Table 2): (1) For gender, 28 (4.1%) were male and 648 (95.9%) were female. (2) There were 231 (34.2%) only children and 445 (65.8%) non-only children in the families of the subjects surveyed. It is common in China for a family to have only one child, which leads to an absence of sibling relationships, often making their childhood socialization processes significantly different from those of non-only children (Stormshak et al., 2009). That is, whether one is an only child is an important demographic variable. Therefore, we believe that only children may have their own unique characteristics in terms of positive emotions, interpersonal assistance, and procrastination behavior. (3) The participants’ residences were rural, 307 (45.4%); township, 191 (28.3%); and urban, 178 (26.3%). Due to the uneven distribution of resources, there can be huge differences in the level of social development, education and healthcare and family income between rural, township and urban areas in China, which are precisely the important factors that affect the physical and mental development of individuals (Hadley, 2003; Propper et al., 2007; Jones et al., 2016). As a result, there may be significant differences in the physical and mental development of higher vocational students from rural, township and urban families, so residence was used as an important demographic variable in this study. (4) The students were enrolled as follows: 236 (34.9%) in the “Nationwide Unified Examination for Admissions to General Universities and Colleges, NUEC,” 267 (39.5%) in the individual examination and 173 (25.6%) in the five-year system (3+2). In China, there are three types of students who enter higher vocational colleges: first, general high school students, who enter higher vocational colleges through the NUEC. Second, students who enter through individual examinations organized at higher vocational colleges, which is also a form of enrollment allowed by the Chinese Ministry of Education. The third is the five-year system, which involves higher vocational colleges and secondary vocational schools cooperating to recruit junior secondary school graduates and provide joint training. In this context, students spend 3 years in a secondary vocational school and then 2 years in a counterpart higher vocational college, also known as the “3+2” system, which is an important form of higher vocational education in China. These three enrollment types differ significantly in terms of the students’ previous learning environments and school teaching styles, all of which influence their students’ learning habits and their initiatives (Davies et al., 2013). Consequently, this study set the origin types of students enrolled as a significant demographic variable. In summary, in this study, gender, only child in family, residence, and origin type were adopted as control variables.

**Table 2:** Descriptive statistics of subjects’ characteristics.

Variables	Types	Frequency	Percentage
Gender	Male	28	4.1
	Female	648	95.9
Only child in family	Yes	231	34.2
	No	445	65.8
Residence	Rural	307	45.4
	Township	191	28.3
	Urban	178	26.3
Origin types	NUEC	236	34.9
	Individual examination	267	39.5
	Five-year system	173	25.6

### 3.3. Data analysis

We used SPSS 26.0 for data analysis and exploratory research. The main statistical analysis methods were as follows: (1) univariate descriptive statistics were used to analyze the current status of higher vocational students’ demographic information, teacher support, procrastination behavior, interpersonal assistance and positive emotions; (2) an independent samples *t* test and one-way ANOVA (Mishra et al., 2019) were used to analyze the variability in the demographic variables of teacher support, procrastination behavior, interpersonal assistance and positive emotions among higher vocational students. (3) Pearson correlation analysis was used to explore the correlations among teacher support, procrastination behavior, interpersonal assistance and positive emotions among higher vocational students. The degree of Pearson correlation is divided into three levels: weak correlation (correlation coefficient of approximately 0.10), moderately strong correlation (approximately 0.30) and strong correlation (≥0.50) (Cohen, 1988). (4) Path analysis is an extension of multiple regression and is a more efficient and direct way of modeling mediators, indirect effects and complex relationships among variables. Path analysis allows for the modeling of structural relationships among observed (latent) variables (Lei and Wu, 2007). Thus, regression-based path analysis was used to determine the covariates and the path coefficients of the multiple mediator model. (5) The bootstrap method was used to conduct a multiple mediator effect analysis using the PROCESS model. The bias-corrected bootstrap method can provide the highest statistical efficacy and the most accurate confidence interval estimates; specifically, the total, direct and indirect effects were considered statistically significant at the 0.05 probability level if the results of the 95% deviation-corrected confidence interval (CI) did not include zero (Hayes, 2018).

## 4. Results

### 4.1. Common method bias test

In general, the adoption of self-report methods for data collection may lead to common method bias effects; therefore, drawing on previous studies (MacKenzie and Podsakoff, 2012), we controlled for common method bias in advance by excluding subjectivity, emphasizing confidentiality, ensuring subject anonymity, and posing positive and negative questions. In addition, after data recovery, all variables were simultaneously subjected to unrotated principal component analysis using the Harman one-way test. The five factors with loadings above 1 were found to explain a cumulative total variance of 68.173%, with the first principal component explaining 27.173% of the variance, not exceeding the threshold value of 40% (Podsakoff et al., 2003). This suggests that there is no serious common method bias in this study.

### 4.2. Comparison of differences among variables

This section examines differences in the demographic dimensions of teacher support, procrastination behavior, interpersonal assistance and positive emotions, which were tested primarily through independent samples *t* tests and one-way ANOVAs (Mishra et al., 2019). We used independent samples *t* tests to compare differences in the means of two groups and one-way ANOVA to compare differences in the means of three or more groups. Notably, if the data met chi-square, we determined whether there was a statistically significant difference among the groups by using the sig value of the one-way ANOVA, followed by a comparison among the groups using the least significant difference method, or LSD, to test for differences among the groups. If the chi-squared was not satisfied, the Welch method was used to determine whether there was a statistically significant difference among the groups, followed by the Tamhane (T2) method of two-by-two comparisons (Dunnett, 1980). As indicated by the results of the test of variance in Table 3, the differences in teacher support, procrastination behavior, interpersonal assistance and positive emotions were not statistically significant in terms of “Gender,” “Only child in family,” “Residence,” or “Origin types.”

**Table 3:** Comparison of differences among variables by demographic dimensions (*N* = 676).

Variables		Teacher support (*M* ± SD)	Procrastination behavior (*M* ± SD)	Interpersonal assistance (*M* ± SD)	Positive emotions (*M* ± SD)
Gender	(1) Male	3.407 ± 0.825	2.364 ± 0.852	3.071 ± 0.930	3.243 ± 0.898
	(2) Female	3.124 ± 0.752	2.854 ± 0.747	3.655 ± 0.791	3.143 ± 0.674
*t*		1.939	−3.377	−3.793	0.759
sig		0.586	0.485	0.378	0.164
Only child in family	(1) Yes	3.122 ± 0.775	2.855 ± 0.786	3.650 ± 0.792	3.158 ± 0.722
	(2) No	3.143 ± 0.748	2.823 ± 0.743	3.621 ± 0.813	3.141 ± 0.664
*t*		−0.347	0.507	0.445	0.296
sig		0.545	0.562	0.799	0.331
Residence	(1) Rural	3.167 ± 0.724	2.785 ± 0.678	3.632 ± 0.762	3.184 ± 0.646
	(2) Township	3.103 ± 0.833	2.831 ± 0.865	3.597 ± 0.847	3.060 ± 0.706
	(3) Urban	3.118 ± 0.729	2.921 ± 0.760	3.666 ± 0.833	3.175 ± 0.720
*F*		0.500	1.955	0.342	2.173
sig		0.607	0.143	0.710	0.115
Hindsight comparison		–	–	–	–
Origin types	(1) NUEC	3.108 ± 0.741	2.892 ± 0.812	3.678 ± 0.867	3.156 ± 0.655
	(2) Individual examination	3.157 ± 0.732	2.822 ± 0.718	3.588 ± 0.797	3.168 ± 0.663
	(3) Five-year system	3.142 ± 0.818	2.775 ± 0.739	3.634 ± 0.729	3.102 ± 0.754
*F*		0.277	1.249	0.732	0.521
sig		0.758	0.288	0.482	0.594
Hindsight comparison		–	–	–	–

### 4.3. Correlation analysis among variables

As shown in Table 4, the higher vocational students did not score very high on the four dimensions, with teacher support (*M* = 3.136), interpersonal assistance (*M* = 3.631), positive emotions (*M* = 3.147), and procrastination behavior (*M* = 2.834) being at the relatively lowest level. In terms of correlations, there was a significant weak positive correlation among gender, procrastination behavior and interpersonal assistance, while there was no significant correlation among gender, teacher support and positive emotions. Simultaneously, there were no significant correlations among only child status, residence, origin type and procrastination behavior, teacher support, interpersonal assistance and positive emotion. At the same time, procrastination behavior was significantly negatively correlated with teacher support, interpersonal assistance and positive emotions, while teacher support, interpersonal assistance and positive emotions were significantly positively correlated. The descriptive statistics and correlations for each of these variables were the basis for in-depth tests of mediating effects.

**Table 4:** Correlation analysis among variables.

No.	Variables	*M*	1	2	3	4	5	6	7	8
1	Gender	1.960	1							
2	Only child in family	1.660	0.085*	1						
3	Residence	1.810	0.042	−0.235**	1					
4	Origin types	1.910	0.110**	−0.018	0.070	1				
5	Teacher support	3.136	−0.074	0.013	−0.030	0.020	1			
6	Procrastination behavior	2.834	0.129**	−0.020	0.072	−0.060	−0.220**	1		
7	Interpersonal assistance	3.631	0.145**	−0.017	0.013	−0.025	0.189**	−0.168**	1	
8	Positive emotions	3.147	−0.029	−0.011	−0.017	−0.028	0.376**	−0.204**	0.347**	1

*p < 0.05, **p < 0.01, ***p < 0.001.

### 4.4. Multiple mediating effects test

Although the correlation between the control and dependent variables is not significant, we believe that gender, only child in family, residence, and origin type are still deserving of attention. According to Becker et al. (2016) and from what we have previously described in Section 3.2, we still set them as control variables to reduce the bias of estimation due to omitted variables. To conduct a more objective analysis, we explored the mediating role of procrastination behavior and interpersonal assistance in the relationship between teacher support and positive emotions among higher vocational students while controlling for gender, only child in family, residence and origin type (Table 5). The results showed that (1) the direct effect of teacher support on positive emotions was significant (*β* = 0.302, *t* = 8.541, *p* < 0.001); (2) teacher support significantly and negatively predicted procrastination behavior (*β* = −0.207, *t* = −5.539, *p* < 0.001), and procrastination behavior also significantly and negatively predicted positive emotions (*β* = −0.088, *t* = −2.473, *p* < 0.01), which suggests that procrastination behavior mediates the relationship between teacher support and positive mood; and (3) teacher support significantly and positively predicted interpersonal assistance (*β* = 0.170, *t* = 4.478, *p* < 0.001), and interpersonal assistance significantly and positively predicted positive emotions (*β* = 0.279, *t* = 7.844, *p* < 0.001), indicating that interpersonal assistance mediated the relationship between teacher support and positive emotions. In addition, procrastination behavior was found to be a significant negative predictor of interpersonal assistance (*β* = −0.161, *t* = −4.202, *p* < 0.001). Therefore, procrastination behavior and interpersonal assistance play a chain mediating role in the relationship between teacher support and the positive emotions of higher vocational students.

**Table 5:** Regression analysis between variables (*N* = 676).

Regression equations		Overall fit index			The significance of regression coefficients	
Outcome variable	Predictor variables	*R*	*R*2	*F*	*β*	*t*
Procrastination behavior	Gender	0.265	0.070	10.129	0.120	3.180**
	Only child in family				−0.014	−0.352
	Residence				0.063	1.639
	Origin types				−0.074	−1.972*
	Teacher support				−0.207	−5.539***
Interpersonal assistance	Gender	0.298	0.089	10.827	0.187	4.952***
	Only child in family				−0.035	−0.924
	Residence				0.018	0.480
	Origin types				−0.061	−1.627
	Procrastination behavior				−0.161	−4.202***
	Teacher support				0.170	4.478***
Positive emotions	Gender	0.480	0.230	28.540	−0.031	−0.889
	Only child in family				−0.011	−0.321
	Residence				−0.004	−0.127
	Origin types				−0.029	−0.830
	Procrastination behavior				−0.088	−2.473**
	Teacher support				0.302	8.541***
	Interpersonal assistance				0.279	7.844***

*p < 0.05, **p < 0.01, ***p < 0.001.

**Figure 1:**
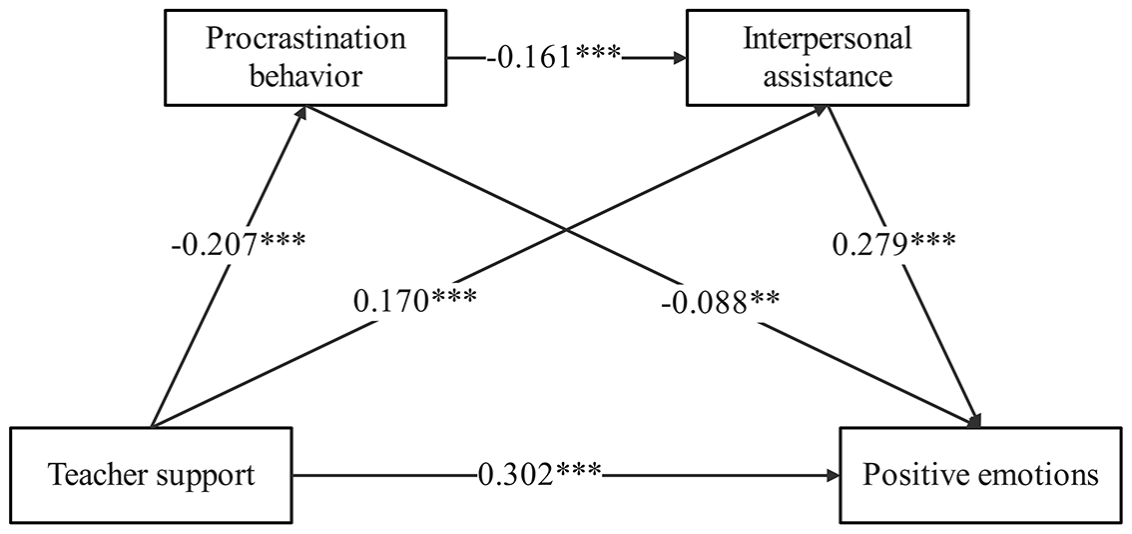
The role of chain intermediaries and the path coefficient.

We further tested for mediating effects using a bias-corrected nonparametric percentage bootstrap method, and the results are shown in Table 6. The bootstrap CI 95% of the total mediating effect did not contain 0 [0.038, 0.094], with an effect value of 0.065, representing 19.11% of the total effect. More specifically, the effect of teacher support on positive emotions was influenced by three mediating chains, all of which reached significant levels. Path 1, consisting of “Teacher Support → Procrastination behavior → Positive Emotions,” had a mediating effect value of 0.018, with a bootstrap 95% confidence interval [0.003, 0.036] that did not contain 0. The mediating effect accounted for 5.29% of the total effect, indicating a significant mediating effect of procrastination behavior. For path 2, consisting of “Teacher support → Interpersonal assistance → Positive emotions,” the mediating effect value was 0.040, and the bootstrap 95% confidence interval [0.019, 0.062] did not contain 0. The mediating effect accounted for 11.76% of the total effect, indicating a significant mediating effect of interpersonal assistance. Path 3: Consisting of “Teacher Support → Procrastination behavior → Interpersonal Assistance → Positive Emotions,” with a mediating effect value of 0.007 and bootstrap 95% confidence interval [0.002, 0.015] that did not contain 0. The mediating effect accounted for 2.06% of the total effect, indicating a significant mediating role of procrastination behavior and interpersonal assistance in the relationship between teacher support and positive emotions. The mediating model and path coefficients for the effect of teacher support on positive emotions among higher vocational students are shown in Figure 1. A two-by-two comparison of the indirect effects of the different pathways to examine whether there were significant path differences revealed that the bootstrap CI 95% for Ind1-Ind2 was [−0.047, 0.006], which contained 0, i.e., the difference was not significant. Similarly, the bootstrap CI 95% for Ind1-Ind3 is [−0.005, 0.027], which also contains 0, and the difference is also not significant. For Ind2-Ind3, the bootstrap CI 95% is [0.010, 0.056], which does not contain 0, and therefore, the difference is significant.

**Table 6:** Mediating effects of procrastination behavior and interpersonal assistance on the relationship between teacher support and positive emotions.

Paths	Effect value	Boot SE	Boot CI 95%		Effectiveness ratio
			Lower limit	Upper limit	
Total indirect effect	0.065	0.015	0.038	0.094	19.11%
Ind1: Teacher support → Procrastination behavior → Positive emotions	0.018	0.009	0.003	0.036	5.29%
Ind2: Teacher support → Interpersonal assistance → Positive emotions	0.040	0.011	0.019	0.062	11.76%
Ind3: Teacher support →Procrastination behavior → Interpersonal assistance → Positive emotions	0.007	0.003	0.002	0.015	2.06%
Ind1-Ind2	−0.022	0.014	−0.047	0.006	–
Ind1-Ind3	0.011	0.008	−0.005	0.027	–
Ind2-Ind3	0.032	0.012	0.010	0.056	–

### 4.5. Comparison of the effects of the three intermediary paths

Teacher support can have an independent mediating effect on positive emotions through procrastination and interpersonal assistance, with a chain of mediating effects between them. In terms of the percentage of mediated effects, among the three mediated paths of teacher support on positive emotion, the differences between Path 1 (Teacher support → Procrastination behavior → Positive emotions) and Path 2 (Teacher support → Interpersonal assistance → Positive emotions) were not significant, while the differences between Path 1 and Path 3 (Teacher support →Procrastination behavior → Interpersonal assistance → Positive emotions) were also not significant. Nevertheless, there was a significant difference between Path 2 and Path 3. This indicates that Path 2 (with an effect ratio of 11.76%) has a stronger mediating effect than Path 3 (with an effect ratio of 2.06%) on the relationship between teacher support and positive emotions. On the other hand, the ratio of the direct effect of teacher support on positive emotions to the total effect was 80.89%. This reveals that increasing teacher support and enhancing interpersonal assistance for higher vocational students may be a more effective way to enhance their positive emotions.

## 5. Discussion and implications

### 5.1. The relationship between teacher support and positive emotions

This study found significant positive correlations among teacher support, interpersonal assistance and positive emotions, indicating that the teacher support (e.g., teachers taking the initiative to help students analyze and solve problems) and interpersonal assistance (e.g., finding classmates, friends, teachers or parents to talk to them when encountering difficulties or unpleasant things) received by higher vocational students in their studies and lives can effectively enhance their positive emotions. Teacher support can be both the recognition and assistance given by teachers in an individual’s learning and life, as well as the positive feedback given to students when they ask for help. From the perspective of ecosystem theory, individuals grow in interaction with their environment, and a good microenvironment promotes optimal development (Bronfenbrenner, 1994). Teacher support can shape a good microenvironment for higher vocational students’ growth. As a form of positive emotional feedback and positive support, teacher support can help higher vocational students adjust their self-regulation strategies so that they can face tasks in life and learning with a positive mindset and have positive emotional experiences. The teacher support derived from this study positively predicted positive emotions, which is also consistent with the results of other studies (Lei et al., 2018). Wang and Eccles (2013) suggested that teacher support can reduce social anxiety and negative affect in high school students and increase their positive emotions. This suggests that teacher support significantly and positively predicts positive emotions, validating Hypothesis 1. In conclusion, it is essential to take actions to strengthen teacher support for higher vocational students to directly affect their positive emotions so that students’ positive psychological capital and mental health can be effectively enhanced. Additionally, teachers in higher vocational colleges can also conduct seminars on emotional awareness experiences, mental health education and group building in classes according to the actual situation, which will increase higher vocational students’ awareness of positive emotions and help them realize the importance of positive emotions. Moreover, higher vocational colleges should provide a supportive educational environment by training teachers on “how to support students” so that teachers can be more targeted in supporting higher vocational students to enhance their positive emotions.

### 5.2. The mediating role of procrastination behavior

From the above results and the practice, various teacher support behaviors (e.g., scholastic support, cognitive support, emotional support, problem solving support, etc.) can boost the confidence of higher vocational students when facing difficult tasks and increase their motivation to solve problems and perform tasks, which in turn will significantly reduce their procrastination behavior. Pychyl et al. (2000) suggested that the absence of teacher support is often a significant factor in student procrastination. Sirois (2004) showed that teacher support and self-efficacy had a negative effect on reducing procrastination. That is, teacher support is crucial for students to successfully address procrastination behavior, and a lack of teacher support will increase the incidence of students’ procrastination behaviors (Codina et al., 2018). Regarding the relationship between procrastination behavior and positive emotions, Sirois et al. (2003) found that procrastination leads to negative effects on a person’s emotions, such as anxiety, stress, and guilt, and these emotions can reduce the level of positive emotions. The above findings provide directional insight into this study, which also found significant negative associations among procrastination behavior, teacher support and positive emotions in our study of higher vocational students in Zhejiang Province, China. More specifically, teacher support significantly and negatively predicted procrastination behavior, while procrastination behavior also significantly and negatively predicted positive emotions. This suggests that procrastination behavior mediates the relationship between teacher support and positive emotions, thus validating Hypothesis 2. This finding revealed the importance of psychometrically screening procrastination behavior in higher vocational students. Moreover, we should take measures to reduce these students’ procrastination behaviors so that their positive emotional experience can be enhanced. More specifically, higher vocational students should learn to manage their time; make specific, feasible and challenging study plans and schedules; and breakdown large tasks into smaller, easily completed tasks to reduce the occurrence of procrastination. In addition, higher vocational students can also reduce their procrastination behavior by means of self-emotional regulation (Diotaiuti et al., 2021). Teachers should provide targeted guidance and support to students to help them remedy their learning deficits, improve their motivation and performance, and thus reduce the occurrence of procrastination.

### 5.3. The mediating role of interpersonal assistance

Student groups with high teacher support have been found to have stronger levels of interpersonal assistance, i.e., teacher support positively predicted interpersonal assistance. This finding is also supported by many existing studies. Pekrun et al. (2009) found that teacher support and encouragement promoted students’ willingness to help others. Caprara et al. (2000) also found significant positive relationships among school social support and teacher support and students’ interpersonal assistance and academic achievement. In addition, it has been shown that interpersonal assistance helps enhance the role of teacher support and further improve students’ positive emotions (Wentzel et al., 2017). By receiving help and support from peers and teachers in learning and social interaction, students are more likely to feel relief from negative emotions and increased self-efficacy (Coffman and Gilligan, 2002). In conclusion, it can be effective to evoke positive emotions in high vocational students by enhancing the mental toughness of delayed groups through effective teacher support. In our research, we found that teacher support positively predicted positive emotions and interpersonal assistance, while high levels of interpersonal assistance could enhance positive emotional experiences, suggesting that interpersonal assistance mediated the relationship between teacher support and positive emotions. In other words, there is a need to strengthen the guidance of teachers’ interpersonal assistance behaviors toward students in the future to effectively improve the psychological resilience of procrastinating student groups, which evokes positive emotions in higher vocational students and in turn ensures learning outcomes and growth quality. The above results validate Hypothesis 3. Therefore, in the future, we need to strengthen the guidance of teachers’ support to effectively enhance the psychological resilience of procrastinating students and evoke their positive emotions, thus ensuring learning effectiveness and growth rate. Meanwhile, higher vocational students should establish positive self-management strategies for their emotions, such as regular exercise, self-talk and seeking support, to reduce procrastination behavior and enhance interpersonal assistance. In practice, teachers should strengthen interpersonal assistance in various ways, such as providing individualized guidance, encouraging mutual support and cooperation among students, and providing help for students to understand and deal with emotional problems. To safeguard the mental health of higher vocational students, future research should further explore an integrated intervention model of procrastination behavior, interpersonal assistance and teacher support for higher vocational students.

### 5.4. The chain mediating role of procrastination behavior and interpersonal assistance

According to Sirois and Pychyl (2013) Short-Term Mood Repair Theory (STMR), procrastination behavior implies a failure of self-regulation. In other words, individuals choose to procrastinate when the need to repair current negative emotions outweighs the need to pursue long-term goals. When faced with challenging or difficult tasks, individuals with high procrastination behavior tend to avoid performing them by “just getting on with it,” a typical form of abandonment. This characteristic is in part an indication of the lack of psychological resilience of high procrastination groups. As a dimension of psychological resilience, interpersonal assistance often has a significant impact on procrastination behavior. In general, the more interpersonal assistance the higher-level student group receives, the less procrastination behavior tends to occur and the more beneficial it is to their mental health and emotional state. More specifically, a high level of interpersonal assistance means the ability to obtain external support through the assistance of others during adverse situations. As a result, they are more comfortable and positive in adverse situations and have higher positive emotional experiences (Philippe et al., 2010). Our study also showed that teacher support negatively predicted procrastination in a group of higher education students and that reduced procrastination behavior in turn increased interpersonal assistance. That is, high levels of interpersonal assistance imply high positive emotional experiences, suggesting that procrastination behavior and interpersonal assistance chain mediate the relationship between teacher support and positive emotions, confirming Hypothesis 4. This suggests that the chain of “procrastination behavior → interpersonal assistance” is also a bridge for teacher support to influence positive emotions in higher vocational students. In other words, teacher support can, in practice, not only work on positive emotions through procrastination behavior and interpersonal assistance alone but also be effective in reducing the procrastination behavior of higher vocational students to increase their level of interpersonal assistance, therefore enhancing their positive emotions. It indicates that we could take measures to simultaneously reduce procrastination behavior and increase interpersonal assistance, which in turn improves the positive emotions of higher vocational students. For example, teachers in higher vocational colleges should focus on students’ emotional needs and encourage diverse learning and exploratory behaviors to enhance their positive emotions and self-efficacy.

## 6. Conclusion

By using a questionnaire survey of 676 higher vocational students in Zhejiang Province, China, this study focused on exploring the relationship between teacher support and their positive emotions and corroborated the direct and indirect effects of teacher support on positive emotions using empirical data. Overall, this study concluded that (1) teacher support significantly and positively predicted positive emotions and that positive feedback, learning assistance and recognition from teacher support enhanced higher vocational students’ positive emotional experiences. (2) Procrastination behavior independently mediates the relationship between teacher support and positive emotions, and there is a path consisting of “Teacher support → Procrastination behavior → Positive emotions,” i.e., a high level of teacher support can reduce the procrastination behavior of higher vocational students, reduce the negative emotions and consequences of procrastination behavior, and thus enhance their positive emotions. (3) Interpersonal assistance mediates the relationship between teacher support and positive emotion, and there is a path consisting of “Teacher support → Interpersonal assistance → Positive emotions.” In other words, high levels of teacher support can enhance interpersonal assistance among higher vocational students, and good interpersonal relationships can significantly improve the experience of positive emotions (especially when they encounter difficulties). (4) Procrastination behavior and interpersonal assistance play a chain mediating role in the relationship between teacher support and positive emotions, and there is a path consisting of “Teacher support → Procrastination behavior → Interpersonal assistance → Positive emotions.” That is, high levels of teacher support increased the level of interpersonal assistance of higher vocational students by reducing procrastination behavior, and higher levels of interpersonal assistance were often associated with better positive emotional experiences.

This study explored the relationships among teacher support, positive emotions, procrastination behavior, and interpersonal assistance and found a positive relationship between teacher support and positive emotions, while procrastination behavior and interpersonal assistance played important roles as mediating variables. Thus, this study provides new insights into educational practice regarding positive emotions. Specifically, we found that teacher support plays an important role in the enhancement of positive emotions by reducing procrastination behaviors and facilitating interpersonal assistance to increase students’ levels of positive emotions. At the same time, procrastination behavior and interpersonal assistance have different mediating roles, which together amplify the relationship between teacher support and positive emotions. These findings can be considered a case study for social support theory, providing a new case and data support for social support theory. In addition, we believe that these findings will support the decision making of administrators in higher vocational colleges in the area of emotion management, which will further improve the effectiveness of their work.

## 7. Study limitations

This study has drawn some valuable conclusions, but there are also two limitations: (1) The subjects in this study were from Zhejiang Province, China, and future studies should further expand the scope of the sample. (2) The relationship between teacher support and positive emotions among higher vocational students was influenced by mediating variables. However, other moderating variables might also be involved, and the stability of the mediating mechanism of action needs to be further examined in the future. These two limitations provide direction for future research, which could lead to a more comprehensive understanding of the mechanisms by which teacher support affects positive emotions.

## Data availability statement

The raw data supporting the conclusions of this article will be made available by the authors, without undue reservation.

## Ethics statement

Ethical review and approval was not required for the study on human participants in accordance with the local legislation and institutional requirements. Written informed consent for participation was not required for this study in accordance with the national legislation and the institutional requirements.

## Author contributions

JW was responsible for data analysis and manuscript writing. JW and CZ were responsible for conceptualization. JW, CZ, QS, and FX were responsible for formal analysis, and all of us were jointly responsible for data coding. All authors contributed to the article and approved the submitted version.

## Funding

This work was supported by the Research Centre for Higher Vocational Education (Tao Xingzhi’s Educational Thought) under the Hangzhou Key Research Base of Philosophy and Social Sciences [grant number 2022JD60].

## Conflict of interest

The authors declare that the research was conducted in the absence of any commercial or financial relationships that could be construed as a potential conflict of interest.

## Publisher’s note

All claims expressed in this article are solely those of the authors and do not necessarily represent those of their affiliated organizations, or those of the publisher, the editors and the reviewers. Any product that may be evaluated in this article, or claim that may be made by its manufacturer, is not guaranteed or endorsed by the publisher.
